# Platelet-Rich Fibrin Facilitates One-Stage Cartilage Repair by Promoting Chondrocytes Viability, Migration, and Matrix Synthesis

**DOI:** 10.3390/ijms21020577

**Published:** 2020-01-16

**Authors:** Chin-Chean Wong, Keng-Liang Ou, Yun-Ho Lin, Ming-Fang Lin, Tsung-Lin Yang, Chih-Hwa Chen, Wing P. Chan

**Affiliations:** 1 Department of Orthopedics, Shuang Ho Hospital, Taipei Medical University, New Taipei City 23561, Taiwan; b8701153@tmu.edu.tw (C.-C.W.); Chihhwachen@gmail.com (C.-H.C.); 2 Department of Orthopedics, School of Medicine, College of Medicine, Taipei Medical University, Taipei 11031, Taiwan; 3 Research Center of Biomedical Devices, Taipei Medical University, Taipei 11031, Taiwan; 4 International Ph.D. Program for Cell Therapy and Regenerative Medicine, College of Medicine, Taipei Medical University, Taipei 11031, Taiwan; 5 Department of Dentistry, Shuang Ho Hospital, Taipei Medical University, New Taipei City 23561, Taiwan; klouyu@gmail.com; 6 Department of Oral Hygiene Care, Ching Kuo Institute of Management and Health, Keelung 20301, Taiwan; 7 Department of Dentistry, Taipei Medical University Hospital, Taipei 11031, Taiwan; 8 School of Dentistry, Health Sciences University of Hokkaido, Hokkaido 061-0293, Japan; 9 3D Global Biotech Inc., New Taipei City 22175, Taiwan; 10 Division of Oral Pathology, Department of Dentistry, Taipei Medical University Hospital, Taipei 11031, Taiwan; kevinyhl@tmu.edu.tw; 11 School of Dentistry, College of Oral Medicine, Taipei Medical University, Taipei 11031, Taiwan; 12 Department of Medical Imaging and Radiological Technology, Yuanpei University, Hsinchu 30015, Taiwan; afun@w.tmu.edu.tw; 13 Department of Radiology, Wan Fang Hospital, Taipei Medical University, Taipei 11696, Taiwan; 14 Department of Otolaryngology, National Taiwan University Hospital and National Taiwan University College of Medicine, Taipei 10051, Taiwan; yangtl@ntu.edu.tw; 15 Graduate Institute of Clinical Medicine, National Taiwan University College of Medicine, Taipei 10051, Taiwan; 16 Research Center for Developmental Biology and Regenerative Medicine, National Taiwan University, Taipei 10051, Taiwan; 17 Department of Medical Research, National Taiwan University Hospital, Taipei 10051, Taiwan; 18 School of Biomedical Engineering, College of Biomedical Engineering, Taipei Medical University, Taipei 11031, Taiwan; 19 School of Medicine, College of Medicine, Taipei Medical University, Taipei 11031, Taiwan; 20 Department of Radiology, School of Medicine, College of Medicine, Taipei Medical University, Taipei 11031, Taiwan; 21 Medical Innovation Development Center, Wan Fang Hospital, Taipei Medical University, Taipei 11696, Taiwan

**Keywords:** autografts, cartilage, chondrocytes, knee, platelet-rich fibrin (PRF)

## Abstract

The main aim of this study is to develop a one-stage method to combine platelet-rich fibrin (PRF) and autologous cartilage autografts for porcine articular cartilage repair. The porcine chondrocytes were treated with different concentrations of PRF-conditioned media and were evaluated for their cell viability and extracellular glycosaminoglycan (GAG) synthesis during six day cultivation. The chemotactic effects of PRF on chondrocytes on undigested cartilage autografts were revealed in explant cultures. For the in vivo part, porcine chondral defects were created at the medial femoral condyles of which were (1) left untreated, (2) implanted with PRF combined with hand-diced cartilage grafts, or (3) implanted with PRF combined with device-diced cartilage grafts. After six months, gross grades, histological, and immunohistochemical analyses were compared. The results showed that PRF promotes the viability and GAG expression of the cultured chondrocytes. Additionally, the PRF-conditioned media induce significant cellular migration and outgrowth of chondrocytes from undigested cartilage grafts. In the in vivo study, gross grading and histological scores showed significantly better outcomes in the treatment groups as compared with controls. Moreover, both treatment groups showed significantly more type II collagen staining and minimal type I collagen staining as compared with controls, indicating more hyaline-like cartilage and less fibrous tissue. In conclusion, PRF enhances the viability, differentiation, and migration of chondrocytes, thus, showing an appealing capacity for cartilage repair. The data altogether provide evidences to confirm the feasibility of a one-stage, culture-free method of combining PRF and cartilage autografts for repairing articular cartilage defects. From translational standpoints, these advantages benefit clinical applications by simplifying and potentiating the efficacy of cartilage autograft transplants.

## 1. Introduction

Cartilage injuries are common in clinical practice, and the rates for routine knee arthroscopic procedures are high [1]. These injuries are disabling and represent major clinical challenges to orthopedic surgeons when a full-thickness cartilage defect eventually progresses to catastrophic arthritis.

Although bone marrow stimulation through microfractures is a simple approach, the outcome is often varied and unpredictable. Moreover, most of the tissue generated is fibrocartilage, a tissue histologically different from that of native joints, which has a hyaline nature [2,3,4,5]. Osteochondral autograft transplantation is a single stage approach in which defects are filled with autologous osteochondral plugs, leading to the formation of homogenous tissue. The disadvantages of this technique include donor-site morbidity, technical difficulties in matching the lesion contour, defect size, and the risk of cartilage and bone collapse [6,7]. In past decades, autologous chondrocyte implantation (ACI) emerged as a suitable technique that might yield hyaline-like regenerative tissues that would have better histological, mechanical, and clinical results than stimulation through microfracture [8,9]. However, mid-term to long-term studies following ACI showed no significant differences as compared with stimulation through microfracture [10,11]. Moreover, ACI requires multiple surgical procedures and complicated in vitro handling which is costly. Most importantly, a sufficient number of chondrocytes could be obtained via monolayer expansion of autologous cells, but this increases the risk of chondrocyte dedifferentiation [12,13].

Tissue engineering and the use of bioactive agents such as growth factors and cytokines are the current methods employed when treating articular cartilage injuries. Many studies confirm the effects of growth factors in promoting cartilage healing [14,15,16,17,18]. Nonetheless, they are not routinely applied in clinical practice because of high costs, short preservation periods, and limited clinical availability. These essential growth factors have been found to release from activated platelets after injury, inflammation, and tissue repair [19,20]. Therefore, preparations of platelet concentrates are viewed as abundant sources of autologous growth factors and serve as alternatives to commercially available products.

Platelet-rich fibrin (PRF) is a second-generation platelet concentrate produced from autologous blood obtained immediately after centrifugation [19,20,21,22]. It can serve as a fibrin biomaterial containing a high concentration of growth factors [21,22,23,24]. No additional activation steps are required, and the release of growth factors from PRF can be steadily sustained [24]. The effects of PRF in facilitating rabbit meniscal and cartilage repairs have been documented in [6,25,26,27] however, the relatively small joint size, thin cartilage layer, and great potential for intrinsic healing have limited the translational value for this one-stage cartilage treatment method.

In this study, we hypothesized that PRF would promote the viability and matrix synthesis of cultured chondrocytes. Moreover, the chemotactic effects of PRF would induce chondrocytes outgrowth, migration, and attachment on the PRF scaffold. In animal disease model, we investigated the repair outcome of this one-stage treatment using PRF combined with autologous cartilage autografts in a clinically relevant large animal model. Finally, by comparing two dicing methods, the question regarding the correlation between cartilage repair outcome and degree of cartilage autograft fragmentation could be answered. From a translational perspective, this novel one-stage cartilage repair method could successfully integrate the advantages of autologous chondrocyte therapy and PRF scaffolds. Meanwhile, no complicated in vitro cell manipulation is needed, rendering the procedure straightforward for future clinical application.

## 2. Results

### 2.1. In Vitro

#### 2.1.1. Dose-Dependent Effect of PRF on Proliferation of Porcine Chondrocytes

**Figure 1:**
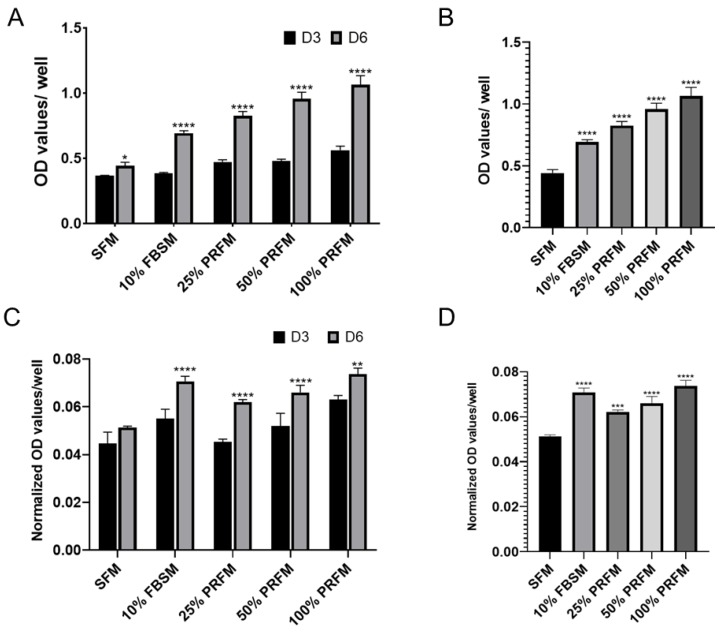
Effects of platelet-rich fibrin conditioned medium (PRFM) on cell viability and accumulated glycosaminoglycan (GAG) content. (**A**) The stimulatory effects of DMEM/F-12 containing 10% fetal bovine serum medium (FBSM), and 25%, 50%, and 100% PRFM on cultured porcine chondrocytes were compared with serum-free medium (SFM) for 6 days. (**B**) On day 6, the cell number significantly increased after FBS and PRF stimulation, in a dose-dependent manner. (**C**) The accumulated GAG content was significantly higher in the groups of cells treated with 10% FBSM, as well as 25%, 50%, and 100% PRFM. (**D**) Chondrocytes treated with FBSM and 25%, 50%, and 100% PRFM for 6 days exhibited significantly higher levels of accumulated GAG content as compared with the control SFM group. Values are presented as mean ± SD. D3, day 3; D6, day 6; and OD, optical density. * *p* < 0.05, ** *p* < 0.01, *** *p* < 0.001, and **** *p* < 0.0001.

To confirm the effect of PRF on promoting cell anabolic activities, chondrocytes were cultured up to six days with either serum free medium (SFM), DMEM/F-12 medium containing 10% fetal bovine serum (10% FBSM), or PRF-conditioned medium (PRFM) in serial diluted concentrations (25%, 50%, and 100%). The cell proliferation rate and accumulated GAG levels were evaluated on day three and six, separately. Cell proliferation was evident after stimulation of porcine chondrocytes with 10% FBSM, as well as 25%, 50%, and 100% PRFM (Figure 1A) The cell number increased significantly from the third to sixth day of culture, particularly in the FBS group and PRFM groups with serial diluted concentrations. On day six, the cell number significantly increased in groups of 10% FBSM (0.716 ± 0.05), 25% PRFM (0.755 ± 0.007), 50% PRFM (0.796 ± 0.06), and 100% PPRFM (0.86 ± 0.07) as compared with SFM (0.49 ± 0.02) (Figure 1B). The data confirmed that different concentrations of PRFM could significantly increase the number of chondrocytes with culture in a dose-dependent manner.

#### 2.1.2. The Effects of PRF on Glycosaminoglycan Matrix Syntheses of Cultured Chondrocytes

No significant increase in accumulated glycosaminoglycan (GAG) levels with time was detected in the SFM group, indicating that serum-deprived chondrocytes lost the ability of synthesizing GAG and in low anabolic status. (Figure 1C) In contrast, chondrocytes cultivated in 10% FBSM, and 25%, 50% and 100% PRFM groups showed increased accumulated GAG expression level from day three to day six, indicating the stimulatory effects of high concentration PRFM were comparable to 10% FBSM. (Figure 1C). On day six, the accumulated GAG content is significantly higher in 10% FBSM and PRFM groups as compared with the control SFM group (Figure 1D). 

#### 2.1.3. Chemotactic Effects of PRF on Cartilage Explants 

**Figure 2:**
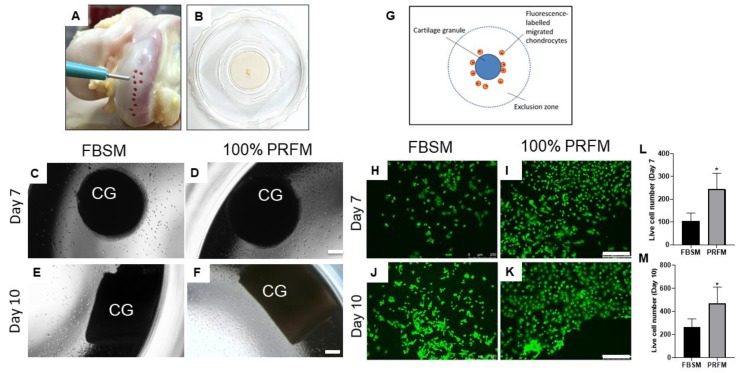
Chemotactic effects of platelet-rich fibrin-conditioned medium (PRFM) on undigested cartilage grafts. (**A**) Cartilage grafts were harvested from the femoral condyle using a skin biopsy punch. (**B**) Cartilage graft was positioned on the well of a confocal dish to allow migratory cells to attach on the stratum of the well. At day 7 (**C**,**D**) and day 10 (**E**,**F**), cellular migration at the tissue level could be observed from the cartilage explants cultured with DMEM/F-12 containing 10% fetal bovine serum medium (FBSM) or 100% PRFM under phase microscopy (scale bar: 100 μm). (**G**) The cell exclusion zone assay is illustrated to show the experimental setting by creating a cell-free central zone for detecting and quantifying fluorescence-labeled live cells. Representative fluorescence images of calcein AM-stained chondrocytes under various culture conditions at day 7 (**H**,**I**) and day 10 (**J**,**K**) (scale bar: 250 μm). Quantification of live cells at day 7 (**L**) and day 10 (**M**) in two groups. The bars indicate the mean ± standard deviation (*n* = 4) for each group. * *p* < 0.05.

Since cartilage grafts are implanted with PRF without enzyme pre digestion, it is crucial to investigate the ex vivo chemotactic effects of PRF on chondrocytes from undigested cartilage grafts. After culture for 7 to 10 days, the phase-contrast micrographs revealed an increasing number of chondrocyte outgrowth from the periphery of the cartilage grafts. At low magnification, the extent of cell migration in 100% PRFM was significantly higher than that in standard 10% FBSM (Figure 2C–F). To further quantify the number of migrated chondrocytes in both groups, the attached cells were fluorescence labeled (Figure 2E–H). The labeled chondrocytes were then counted using Image J analytical software. The number of living cells in the 10% FBSM group at day seven was lower (103.5 ± 35.8) as compared with the 100% PRFM group (264.5 ± 71.5, *p* = 0.01, Figure 2L). After culture for 10 days, an increased number of cells migrated and attached in both the 10% FBSM (244.5 ± 69.9) and 100% PRFM (470.7 ± 140.2) groups. Additionally, the 100% PRFM group had significantly more viable cells as compared with the 10% FBSM group (*p* = 0.04, Figure 2M).

#### 2.1.4. Cellular Outgrowth from Cartilage Grafts on PRF Scaffolds

**Figure 3:**
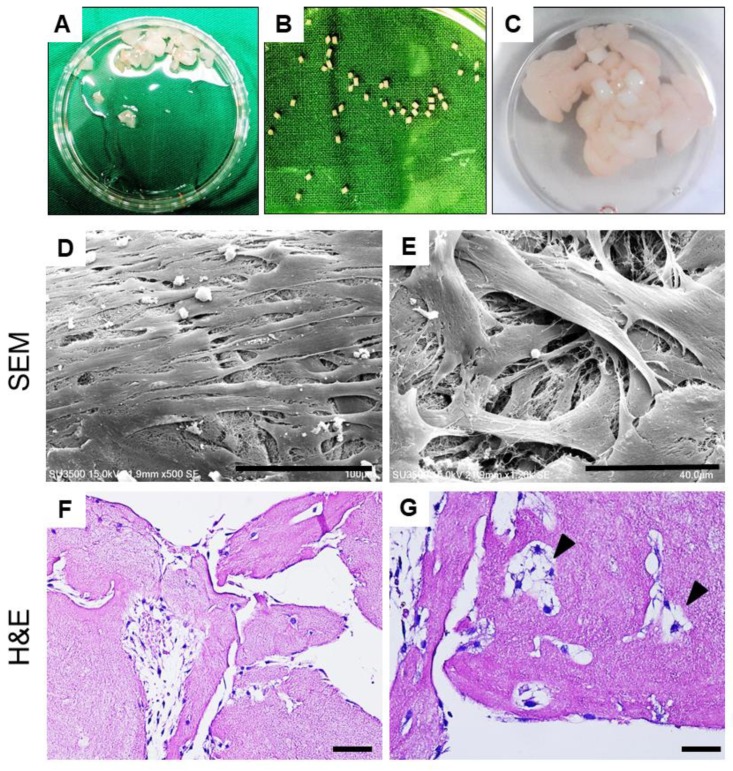
(**A**–**C**) Illustration of PRF-cartilage graft co-culture model: (**A**) Diced platelet-rich fibrin, (**B**) cartilage grafts harvested from porcine joint, and (**C**) co-culture of platelet-rich fibrin and cartilage grafts. (**D**,**E**) Scanning electron microscopy (SEM) observation of the PRF scaffold after 10 days of culture. The SEM images showed chondrocytes attachment and growth on PRF from low to high magnifications. (**F**,**G**) Hematoxylin and eosin (**H**,**E**) of histological sections of PRF scaffold on day 10 showed the chondrocyte migration and growth on porous structures. At magnified view, chondrocytes were in cluster and presented with lacunae-like structures (arrowheads). Scale bars: 100 μm (**D**), 40 μm (**E**), 50 μm (**F**), and 25 μm (**G**).

The illustration of PRF-cartilage grafts co-culture model is shown in Figure 3A–C. After culture for 10 days, images obtained using SEM revealed notable chondrocyte outgrowth from cartilage grafts. At low magnification, the migratory chondrocytes were found firmly attaching onto the PRF scaffold, indicating good cell-scaffold affinity. At high magnification, the cells were polygonal in shape and showed extended cytoskeletons and pseudopodia (Figure 3D,E). The hematoxylin and eosin (H&E) staining of PRF scaffold showed plentiful migrated chondrocytes attached on PRF scaffold (Figure 3F). The magnified views also showed the architecture of PRF scaffold with chondrocytes growing inside. Moreover, the chondrocytes were in cluster with lacunae-like structures (arrowheads, Figure 3G).

### 2.2. In Vivo

#### 2.2.1. Macroscopic Assessments of Specimens

**Figure 4:**
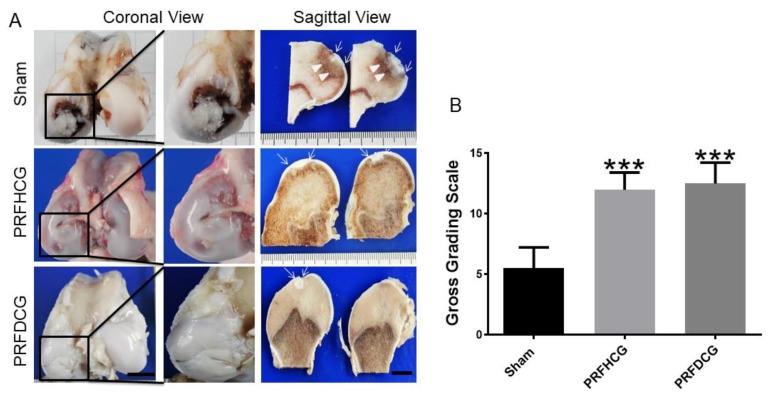
Gross view and grading scale of repaired regions: (**A**) Macroscopic coronal (left column) and sagittal (right column) views of representative defects from each of three experimental groups at 6 months (scale bar: 10 mm). Insets show magnified coronal views of the cartilage defect regions of representative specimens from each group and (**B**) detailed quantitative gross grading scale. The bars indicate the mean ± standard deviation (*n* = 4) for each group. *** *p* < 0.001. PRFHCG, platelet-rich fibrin with hand-diced cartilage grafts and PRFDCG, platelet-rich fibrin with device-diced cartilage grafts.

Mean gross grading was performed based on defect coverage, neocartilage color, defect margin, and surface smoothness. Six months after operation, cartilage regeneration was better in the groups implanted with platelet-rich fibrin with hand-diced cartilage grafts (PRFHCG) and device-diced cartilage grafts (PRFDCG) than in controls. Grossly, the defects were filled with reparative tissue, and the condylar articular surfaces were intact, smooth, and hyaline-like, resembling normal articular tissue. Implants of the PRF and cartilage grafts were integrated with adjacent normal tissues, and boundaries between implanted and native tissues were unclear. The repair tissues were white and mostly even with the surrounding cartilage, although some fissures and small depressed areas were observed at the periphery of the defects (Figure 4A). In contrast, the untreated controls showed very limited repair in the defect area, leaving a remarkable central depressed region. Grossly, the articular condyle was coronally concave and had a non-uniform surface over the defect site. Cartilage tissue was scarcely detectable. In the sagittal view, subchondral bone beneath the cartilage defect in controls presented with edematous change, suggesting weight overload. Quantitatively, the macroscopic scores for the PRFHCG group (12 ± 1.4) and PRFDCG group (12.5 ± 1.7) were significantly higher than that for controls (5.5 ± 1.7) (Figure 4B). Additionally, no significance difference between these treatment groups was noted (*p* = 0.90).

#### 2.2.2. Histological Evaluation of Defect Healing

**Figure 5:**
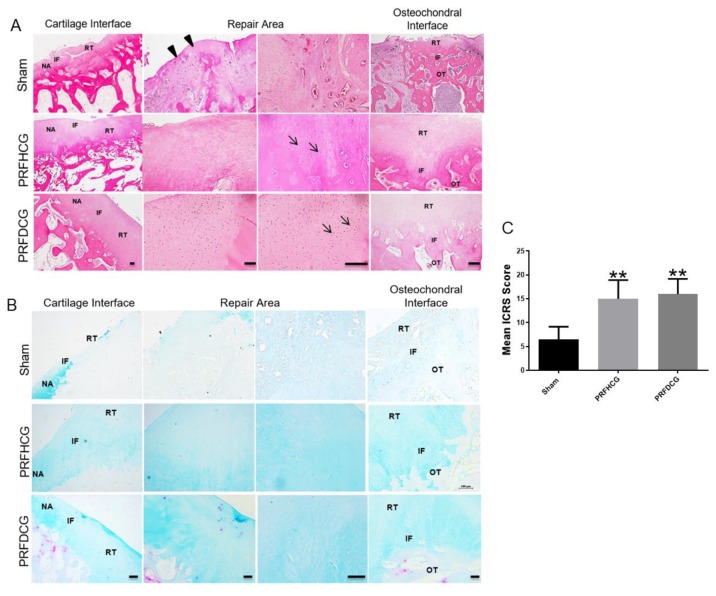
(**A**) Hematoxylin and eosin (H&E) staining of histological sections of representative defects from the control group (upper panel) and the groups treated with platelet-rich fibrin and either hand-diced cartilage grafts (PRFHCG group, middle panel) or device-diced grafts (PRFDCG group, lower panel). In untreated controls, the defect was partially filled with fibrous-like repair tissue (arrowheads). In the treatment groups, the cartilage surfaces were smooth and continuous, indicating satisfactory interface integration between the implanted cartilage grafts and native cartilage. The defects were covered with implanted cartilage grafts, resulting in newly grown repair tissues with mature lacuna structures (arrows). Scale bars: 100 μm. (**B**) Alcian blue staining of histological sections of representative defects of the three groups. Scale bars: 100 μm. NA, native area; IF, interface; RT, repaired tissue; and OT, subchondral bone. (**C**) Mean International Cartilage Repair Society scores evaluating repair tissues in the control, PRFHCG, and PRFDCG groups at 6 months. The bars indicate the mean ± standard deviation (*n* = 4) for each group. ** *p* < 0.01.

The histological pictures of each group were specifically classified into the following three distinct regions: the cartilage interface, the repair area, and the osteochondral interface. On average, high cellularity at the defect site and a certain amount of inflammatory fibrous tissue were seen around the defects in controls. This group also showed a significantly greater inflammatory response in the subchondral bone (Figure 5A, upper panel). In contrast, the regenerated articular cartilage surfaces in the treatment groups were smooth and continuous which indicated that cartilage repair was relatively complete (Figure 5A, middle and lower panels). In both PRFHCG and PRFDCG groups, repair tissue integrated with contiguous native tissue and the subchondral bone. At the greater magnification levels, chondrocytes in regenerated cartilage were found to be generally viable in both treatment groups. Although the repair outcome in treatment groups were similar, there were some differences existed between PRFHCG and PRFDCG groups. In PRFDCG group, most of the chondrocytes in the repair area were oval shaped with lacunae structure. Moreover, the chondrocytes in the PRFDCG group were more evenly distributed in repair area than in PRFHCG group. However, the distribution of chondrocytes in both treatment groups lacked a normal zonal structure which indicated incomplete remodeling. Figure 5B shows the repaired regions stained with alcian blue (AB). These results further support the H&E observations. Repaired regions in the controls consisted primarily of fibrous tissue that was negatively stained for AB. The International Cartilage Repair Society (ICRS) histological scores at six months reflected good to excellent results for the PRFHCG (15 ± 3.9) and PRFDCG (16 ± 3.2) groups as compared with controls (6.5 ± 2.6, Figure 5C). Finally, despite differences in cellular distribution and tissue homogeneity, no significant differences in histological performances were found between the treatment groups (*p* = 0.90).

#### 2.2.3. Chondrogenic Immunohistochemical Analysis

**Figure 6:**
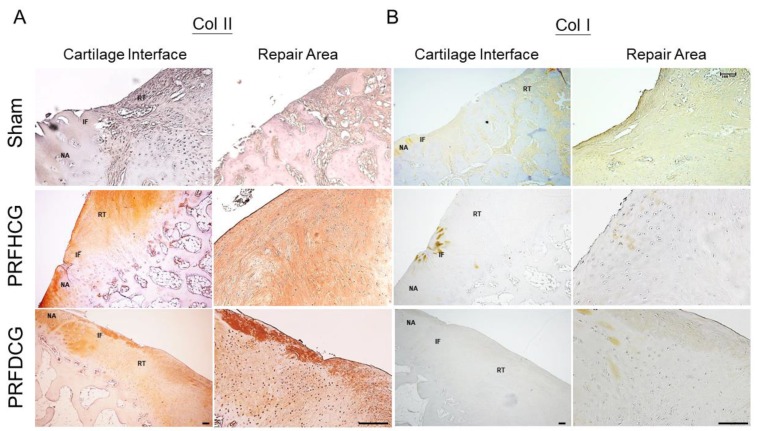
Immunostaining of the repair tissue by antibodies to collagen type II (Col II) and collagen type I (Col I). (**A**) In PRFHCG and PRFDCG groups, the repair areas were stained positively for Col II (brown stained region) but negatively for Col I. (**B**) In control group, the repair area was mainly composed of fibrous tissues that were positively stained for Col I (light brown stained region). Scale bar: 100 μm.

Immunohistochemical staining using Collagen I (Col I) and II (Col II) showed that most of the repair tissue in the two treatment groups stained positively for Col II but negatively for Col I (Figure 6). These results indicate that repair tissues in the treatment groups showed features of hyaline-like cartilage. In the control group, the unhealed chondral defect led to the formation of a central depressed area filled with heterogeneous immature tissue that was negative for Col II and positive for Col I, suggesting a fibrous nature.

## 3. Discussion

This study reveals the potential of PRF in the development of an autologous, single stage, and culture-free therapeutic approach for critically sized chondral defects in a clinically relevant animal model. First of all, the study revealed the anabolic and chemotactic effects of PRF on chondrocytes at both cellular and tissue levels, which could translate into a viable strategy for in vivo cartilage repair. This treatment strategy is distinct from previous methods in cartilage repair because it relies on the bioactive PRF scaffold to attract and nourish chondrocytes from undigested cartilage grafts. On the basis of our results, PRF could induce cell migration and serve as a niche in promoting cell viability and differentiation. 

The presence of appropriate cell types and sufficient chondrocyte numbers are essential to initiate cartilage tissue healing. The PRF scaffold acts as a reservoir of growth factors and cytokine. The molecules essential for chondral repair, such as TGF-β, PDGF, and IGF-1, are entrapped within interconnecting PRF fibrin meshwork [14,15,16,17,18]. Many previous studies have revealed the stimulatory effects of these bioactive molecules on chondrocytes viability, proliferation, and cartilaginous matrix production [21,22,23,24]. Our results showed that porcine chondrocytes treated with proportionally increased concentrations of PRFM demonstrated increased cell viability in a dose-dependent manner (Figure 1B). In fact, the proliferative effect of PRF on porcine chondrocytes, in this current study, is in line with our previous works using rabbit chondrocytes [27]. Moreover, the results are also compatible with other PRP-related in vitro studies with bovine, sheep, or human chondrocytes [28,29,30,31]. In addition, synthesis of extracellular matrices specific to chondrocytes is essential to establishing the microenvironment for cartilage repair and regeneration. Aggregated proteoglycan is one the major extracellular components of articular cartilage consisting of a core protein with numerous covalently attached GAG chains. Our data showed that PRFM has similar effect in promoting porcine chondrocytes GAG synthesis as 10% FBSM during six day cultivation, confirming the potential of PRF for cartilage repair (Figure 1C,D). 

The bioactivity and chemotactic effect of PRF on cell recruitment and migration is crucial to the development of our one-stage, culture-free, and scaffold-based approach. In previous studies, the efficacy of PRF chemotaxis at both the cellular and tissue levels has been investigated, showing that PRF can promote rabbit meniscocyte and chondrocyte migration, similar to that seen when using platelet-rich plasma [25,27,32]. These results were also consistent with studies performed on rabbit and goat models [6,33]. In this study, the chemotactic effects of PRF on undigested cartilage grafts were first evaluated using an explant culture model. The results revealed that 100% PRFM can induce significantly more chondrocyte outgrowth than 10% FBSM during 10 day cultivation. In addition, an ex vivo co-culture model mimicking in vivo microenvironment also confirmed the ability of cellular migration in the tissue level. The SEM and histological results not only displayed notable chondrocytes outgrowth from cartilage grafts and successful attachment onto the porous PRF scaffold, but also significant cellular ingrowth into PRF porous structure. We believe that these data lend supports to the PRF role as a bioactive scaffold to facilitate chondrocyte outgrowth, migration, and attachment, which would benefit the one-stage therapeutic approach for cartilage repair. 

On the basis of the results, it is reasonably to believe that diced cartilage grafts, when implanted with bioactive PRF scaffolds in vivo, were expected to show similar chondrocyte migration and subsequent tissue healing. The overall histological appearance of the treatment groups at six months showed hyaline-like cartilage healing. The repaired region in PRFHCG and PRFDCG groups exhibited high cellular viability, abundant GAG/proteoglycan syntheses, and increased expressions of articular cartilage marker (Col II). Results clearly indicate that diced cartilage grafts played a key role in the PRF composite as a source of chondrocytes and conditioning factors during the healing process. The superior healing found in the treatment groups, however, lacked the overall zonal organization of hyaline cartilage, and some areas of incomplete repair were noted, implying the technique needs improvement. On the one hand, the histological changes of subchondral bone in each group were examined both macroscopically and microscopically (Figure 4 and Figure 5). The results showed that significant reactive changes (bone overgrowth, edema) secondary to stress concentration and overload were detected in subchondral bone region in controls. On the other hand, these reactive phenomena were absent in PRFHCG and PRFDCG indicating that the functionality of the repair tissue was sufficient and nearly complete. 

In 2015, Bonasia et al reported that the degree of chondral fragmentation affected extracelullar matrix (ECM) production [34]. That study showed that ECM synthesis was inversely proportional to chondral fragment size [34]. However, limited studies have disclosed the relationship between the degree of cartilage graft fragmentation and cartilage repair outcomes in an in vivo study. In this study, a cartilage-dicing device was used to yield cuboid-shaped cartilage grafts that were of a standard size (1 mm per side). The surface-area-to-volume ratio is inversely proportional to size. Therefore, chondrocytes residing inside smaller cartilage grafts would interact with PRF more effectively because of the larger surface area provided. This results in a larger number of chondrocytes available to migrate and participate in in situ tissue repair.

There are a few limitations in this study. First, the mechanical properties of the repaired cartilage were not measured. However, compression testing techniques would be difficult to apply because of the curvature in the cartilage surface and the irregular shape of the repaired cartilage. According to the literature, evaluation six months postoperatively could be adequate for proof-of-concept. Nevertheless, a larger animal sample size with a longer study period should be conducted to further evaluate the efficacy and long-term outcome of this single stage therapeutic approach for cartilage repair.

## 4. Materials and Methods

### 4.1. Study Design and Ethics Statement

All tissue harvest and surgical procedures on experimental animals were carried out according to the guide for the Care and Use of Laboratory Animals and were approved by the Institutional Animal Care and Use Committee (IACUC approval P102005, on 25 Jan 2013 and PIG-108012, on 21 June 2019). 

### 4.2. Preparation of PRF and PRF-Conditioned Medium

The PRF was prepared following the process described by Choukroun et al. [21] During surgery in the operating room, 8 mL whole blood from the jugular vein was collected in a plastic Vacuette^®^ tube via venipuncture. Immediate centrifugation of the Vacuette^®^ tube at 3000 rpm for 10 min in a tabletop centrifuge (Digisystem, Laboratory Instruments Inc., New Taipei City, Taiwan) resulted in separation into the following 3 layers: red blood cells (bottom layer), acellular plasma (top layer), and PRF. The PRFM was prepared by soaking a PRF gel in 10 mL serum free Dulbecco’s modified Eagle’s medium (DMEM/ F-12; Gibco, Paisley, UK) in a centrifuge tube (defined as 100% PRF-conditioned medium). The tubes were then put into a tube rotator placed at 4 °C for 24 h. The conditioned medium was, then, collected, filtered, and diluted to 50% and 25% proportionally. 

### 4.3. Harvest of Porcine Cartilage Explants

Three stifle joints of 6-month-old miniature pigs were obtained from Pigmodel Animal Technology Co., Ltd (Miaoli, Taiwan). The specimens were washed, skinned, and opened under sterile conditions within 3 h of slaughter. Before harvesting, the 1 mm skin biopsy punch (Kai Medical, Tokyo, Japan) was first marked at 1 mm in depth. Then, full-depth and standard-sized articular cartilage explants were acquired from the femoral condyle. The harvested cartilage specimens were cultured in DMEM/F-12 containing 10% of FBS.

### 4.4. Isolation and Cultivation of Porcine Chondrocytes 

The porcine cartilage explants were then diced into small fragments and digested with 0.05% (*w*/*v*) collagenase II (Gibco, Paisley, UK) for 18 h at 37 °C. The digestion suspension was washed by centrifugation (5 min at 1200 rpm) in DMEM/F-12 medium containing 10% FBS. Cells were plated at a density of 5 × 10^5^ cells/10 cm dish in 10 mL of culture medium. The cells were, then, cultured in a 5% CO_2_, and 90% humidity incubator at 37 °C for several days until confluence was reached. The medium changed every 3~4 days. When the cells reached 80% confluence, they were trypsinized and transferred into new 10 cm dishes with a density of 5 × 10^5^ cells/dish. 

### 4.5. Effects of PRF on Chondrocytes Viability

The P1 chondrocytes were suspended in DMEM/F12 medium at a density of 1 × 10^4^ cells/mL per well and loaded on 24-well plate. After 24 h, the medium was removed and 1 mL of either serum free medium (SFM), 10% FBS medium (FBSM), 100%, 50%, or 25% PRF conditioned medium (PRFM) were added to respective wells. The medium was changed every 3 days, for a total culture period of 6 days. To further evaluate the effects of PRF on cell proliferation, at each time point (day 3 and 6), the viable cell number in the respective groups was counted using thiazolyl blue tetrazolium bromide (MTT, Invitrogen, Carlsbad, CA, USA) following the manufacturer’s instructions. Briefly, MTT reagent was added to each sample and incubated for 3 h to allow the formation of MTT formazan. The resulting formazan was educed with dimethyl sulfoxide (DMSO, Sigma-Aldrich, Inc., St. Louis, MO, USA), and the absorbance of each solution was measured at a wavelength of 595 nm with a microplate reader (Bio-Rad, Hercules, CA, USA) in quadruplicate.

### 4.6. PRF on Chondrocytes Glycosaminoglycan Synthesis

The accumulated GAG level was measured via Alcian blue staining. Briefly, the cells were fixed with 10% formaldehyde for at least 30 min, rinsed with distilled water followed by incubated in 0.0018 M H_2_SO_4_ for 30 min. Then, the acid solution was removed completely before adding Alcian blue solution (1% Alcian blue 8GX in 0.0018 M H_2_SO_4_) (Sigma-Aldrich). The staining step took 3 h, followed immediately by washing with 0.018 M H_2_SO_4_ for another 3 h to remove redundant dye. Finally, the bound dye was eluted with dissociation buffer (4 M guanidine hydrochloride in 33% 1-propanol with 0.25% Triton X-100) (Sigma-Aldrich). The absorbance of each sample was then measured at 600 nm using a microplate reader in quadruplicate.

### 4.7. Ex Vivo Model of Chondrocytes Chemotaxis

A custom-made cell exclusion zone assay was prepared. A cartilage graft was placed in the center well of a 3 cm confocal dish. The graft was then cultured with either 10% FBSM or 100% PRFM. To secure the cartilage graft to the base of the well and to reduce excessive micromotion, a cover glass was placed atop the well. Chondrocyte outgrowth from the grafts were contained and eventually attached to the bottom of the well, allowing observation using an inverted light microscope (Olympus, Tokyo, Japan) on the seventh and tenth days of culture.

### 4.8. PRF Chemotactic Effects on Chondrocytes Outgrowth and Viability

Detection and quantitation of migrated chondrocytes from tissues were evaluated on days 7 and 10. The chondrocytes were incubated with cell viability probes calcein acetoxymethyl and ethidium homodimer (Thermo Fisher Scientific, Waltham, MA, USA), labeling living chondrocytes green and dead chondrocytes red. The live cells were observed under a fluorescence microscope, and images were captured and analyzed using image analyzing software (Image J, National Institutes of Health, Bethesda, Rockville, MD, USA).

### 4.9. Co-Culture of PRF Scaffolds with Cartilage Grafts

An ex vivo exploration of the chemotactic effects of PRF on chondrocytes residing in undigested cartilage grafts was then performed. Five cartilage explants (each measuring 1 cubic millimeter) were co-cultured with 1 cc PRF scaffolds in a 3 cm confocal dish, allowing the formation of PRF-cartilage grafts. The central well of the confocal dish confined the construct and prevented it from scattering while the media was changed. Chondrocyte migration and cellular outgrowth on the PRF scaffold was evaluated after 10 days of incubation. For SEM, the PRF scaffold was fixed in 2.5% glutaraldehyde for 1 h and treated for desiccation. The specimens were then sputter coated with 20 nm of gold and, subsequently, examined with an SEM. Photographs were taken at 15 to 25 kV with 15 to 20,000 magnifications. For histological analysis, the PRF scaffold was fixed in 10% buffered formalin for at least 48 h and stained with hematoxylin and eosin (H&E).

### 4.10. Surgical Procedure and Cartilage Dicing Process

**Figure 7:**
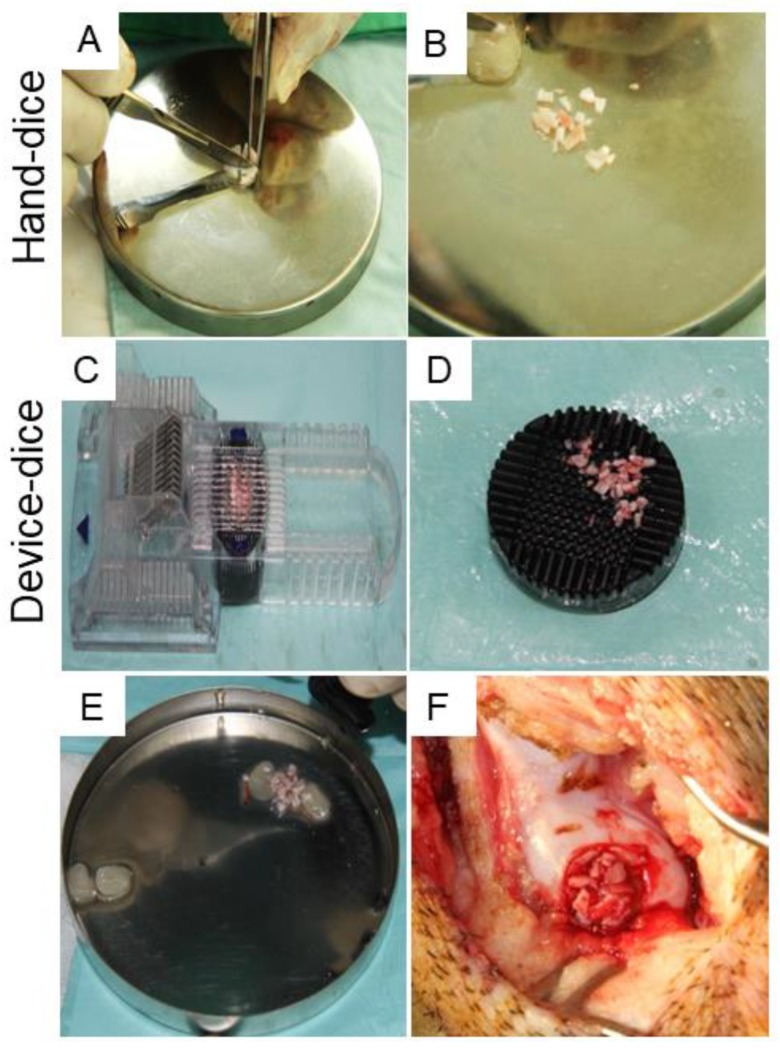
A full-thickness articular cartilage defect was created at the medial femoral condyle, then the harvested graft was processed into cartilage grafts using either the hand-diced method (**A**,**B**) or a device-diced method that employs a custom-made cartilage grinder (**C**,**D**). (**E**,**F**) After mixing with PRF, the diced cartilage grafts were implanted into the defects.

Twelve hybrid pigs (aged 3 to 6 months and 65 to 69 kg) were used. The animals were sedated using 3 to 4 mg/kg azeperonum (Stresnil, 40 mg/mL; Janssen Pharmaceutical N.V., Beerse, Belgium) and atropine (Tai Yu, Taipei, Taiwan) intramuscularly. Full-thickness cartilage defects (8 mm in diameter and 1 to 1.5 mm in depth) were created in the medial femoral condyle to mimic articular cartilage damage from physical overload or stress. Briefly, a trephine and curette were used to remove cartilage flaps. Then, the removed tissues were used as autologous cartilage grafts, prepared using a dicing process before implant. In the controls, the defects were left untreated. In one treatment group, the PRFHCG group, PRF was mixed with autologous cartilage grafts prepared by hand dicing the harvested cartilage into small fragments (Figure 7A,B). Because the cartilage graft was cut manually, the shapes and sizes of the grafts were inconsistent, ranging from 1 to 3 mm in diameter. In the other treatment group, the PRFDCG group, a custom-made cartilage grinding device was used to standardize cartilage granule preparation as well as minimize interoperator variation, providing cuboid-shaped (1 mm per side) grafts which were mixed with PRF and used to treat the lesions (Figure 7C,D). Digestive enzymes and biological manipulation were not used. In both treatment groups, each defect was filled with 0.75 cc PRF and 0.25 cc cartilage grafts (Figure 7E,F).

After graft implantation, a periosteal patch was placed on top of the defect to retain the cartilage filling, and the patch was fixed with absorbable sutures. After completion of the procedure, the patella was returned to its normal position, and the joint capsule, subcutaneous tissues, and skin were sutured to close the wound. Postoperatively, penicillin (40,000 IU/kg for 5 days) and ketoprofen (2.2 mg/kg for 3 days) were administered.

### 4.11. Outcome Measurement and Gross Grading

After 6 months, all animals were euthanized by an intravenous overdose of thiamylal. Stifle joints were harvested within 1 h and stored at 4 °C until gross grades could be assessed. Once the joint was completely exposed, digital photographs of the defect area were taken. They were, subsequently, used to grade the gross appearance of the articular cartilage according to the method described by Wayne et al. [35] for macroscopic assessment of cartilage repair tissue (Supplementary Table S1). Following macroscopic assessment, each femoral condyle was fixed in 10% buffered neutral formalin, decalcified, and embedded in paraffin for routine histological sectioning.

### 4.12. Histological and Immunohistochemical Staining

All specimens were fixed in 10% buffered formalin for at least 3 days, decalcified in 5% nitric acid, embedded in paraffin, and sectioned. Sections (5 mm) were stained with hematoxylin and eosin (H&E), with Alcian blue (AB), and with primary antibodies to type I collagen (Col I: BS-0578R, 1:50 dilution, Bioss, Woburn, MA, USA) and type II collagen (Col II: BS-0709R, 1:100 dilution, Bioss). The H&E histological stain was used to evaluate the integrity of the cartilage interface and integration of the repair tissue. Moreover, the cellularity, collagen alignment, cell morphology, and overall organization of the repaired tissues were revealed. Alcian blue and immunohistochemical staining were used to assess the quality of the repaired tissue by characterizing the type, amount of synthesis, and distribution of regenerated extracellular matrices (ECMs).

### 4.13. Histological Assessment

Using the H&E stained sections, regenerated cartilage was scored based on the International Cartilage Repair Society (ICRS) Visual Histological Assessment Scale (supplementary Table S2) [36]. Specimens were evaluated independently by 3 individual investigators who were blinded to the treatment assignment and numerical data. The score for each parameter was calculated by averaging the 3 scores.

### 4.14. Statistical Analysis

Statistical analysis was performed using Graph Pad Prism (v7.01, GraphPad, San Diego, CA, USA). When histological scores were compared between treatment groups, one-way analysis of variance was utilized followed by the multiple comparisons Scheffé test. The Dunnett test was used when histological scores were compared between the control and treatment groups. Differences were considered significant when *p* < 0.05. Means and confidence intervals were calculated from quantitative data.

## 5. Conclusions

In conclusion, this study suggests that combining PRF with diced autologous cartilage grafts can enhance cartilage repair at the defect site, and therefore can increase the benefits of a one-step treatment. On the basis of these results, a novel one-stage therapeutic approach to potentiate the application of autologous cartilage grafts is expected to expedite the clinical translation of cartilage repair.

## Supplementary Materials

Supplementary materials can be found at https://www.mdpi.com/1422-0067/21/2/577/s1.
