# Supplementary material for: Platelet-Rich Fibrin Facilitates One-Stage Cartilage Repair by Promoting Chondrocytes Viability, Migration, and Matrix Synthesis
Source: Int J Mol Sci. 2020 Jan 16;21(2):577. doi: 10.3390/ijms21020577 (PMC7014470; doi:10.3390/ijms21020577)
Supplement: Supplementary file 1 [file ijms-21-00577-s001.zip › ijms-687166-SI.pdf]

Supplementary Materials

Supplementary Table S1. Scoring System Used for Gross Assessment and Quantitation of Porcine Cartilage Repair.

Gross Appearance	Grade
**Coverage**	
>75% fill	4
50–75% fill	3
25–50% fill	2
< 25% fill	1
No fill	0
Neocartilage color	
Normal	4
25% yellow/brown	3
50% yellow/brown	2
75% yellow/brown	1
100% yellow/brown	0
Defect margins	
Invisible	4
25% circumference visible	3
50% circumference visible	2
75% circumference visible	1
Entire circumference visible	0
Surface smoothness	
Smooth/level with normal	4
Smooth but raised	3
Irregular 25–50%	2
Irregular 50–75%	1
Irregular >75%	0

Supplementary Table S2. Scoring System of International Cartilage Repair Society Used for Histological Assessment and Quantitation of Porcine Cartilage Repair

Feature		Score
I. Surface		
	Smooth/ continuous	3
	Discontinuities/irregularities	0
II. Matrix		
	Hyaline	3
	Mixture: Hyaline/fibrocartilage	2
	Fibrocartilage	1
	Fibrous tissue	0
III. Cell distribution		
	Columnar	3

	Mixed: Columnar/cluster	2
	Cluster	1
	Individual cells/disorganized	0
IV. Cell population		
	Predominantly viable	3
	Partially viable	1
	<10% viable	0
V. Subchondral bone		
	Normal	3
	Increased remodeling	2
	Bone necrosis/granulation tissue	1
	Detached/fracture/cells at base	0
VI. Cartilage mineralization (calcified cartilage)		
	Normal	3
	Abnormal/inappropriate	0
